# Pretty crowds are happy crowds: the influence of attractiveness on mood perception

**DOI:** 10.1007/s00426-020-01360-x

**Published:** 2020-05-25

**Authors:** Alica Mertens, Johanna Hepp, Andreas Voss, Amelie Hische

**Affiliations:** 1 Institute of Psychology, Heidelberg University, Hauptstr. 47-51, 69117 Heidelberg, Germany; 2 Department of Psychosomatic Medicine, Central Institute of Mental Health, Medical Faculty Mannheim/Heidelberg University, Mannheim, Germany

## Abstract

Empirical findings predominantly support a happiness superiority effect in visual search and emotion categorization paradigms and reveal that social cues, like sex and race, moderate this advantage. A more recent study showed that the facial attribute attractiveness also influences the accuracy and speed of emotion perception. In the current study, we investigated whether the influence of attractiveness on emotion perception translates into a more general evaluation of moods when more than one emotional target is presented. In two experiments, we used the mood-of-the-crowd (MoC) task to investigate whether attractive crowds are perceived more positively compared to less attractive crowds. The task was to decide whether an array of faces included more angry or more happy faces. Furthermore, we recorded gaze movements to test the assumption that fixations on happy expressions occur more often in attractive crowds. Thirty-four participants took part in experiment 1 as well as in experiment 2. In both experiments, crowds presenting attractive faces were judged as being happy more frequently whereas the reverse pattern was found for unattractive crowds of faces. Moreover, participants were faster and more accurate when evaluating attractive crowds containing more happy faces as well as when judging unattractive crowds composed of more angry expressions. Additionally, in experiment 1, there were more fixations on happy compared to angry expressions in attractive crowds. Overall, the present findings support the assumption that attractiveness moderates emotion perception.

## Introduction

The fast and correct identification of emotional expressions in human faces is essential for social interactions, because facial expressions signal a person’s potential intentions and behavior (Calvo & Marrero, 2009). While a smiling face signals affiliation and approachability, an angry face signals a lack of approachability or even threat of aggression (Scherer & Wallbott, 1994). Due to the centrality of facial emotion perception for adaptive social interaction, many theorists have argued that humans can automatically detect signs of affiliation or threats of aggression in faces (e.g., Öhman & Mineka, 2001). Building on this assumption, some have postulated that specific emotional expressions have a perception advantage over others. Initially, a perception advantage was postulated for angry faces (Hansen & Hansen, 1988; Öhman, Lundqvist, & Esteves, 2001; Pinkham, Griffin, Baron, Sasson, & Gur, 2010), but recent studies have found compelling evidence for an advantage for happy faces, termed “happiness superiority effect” (Becker, Anderson, Mortensen, Neufeld, & Neel, 2011; Savage, Lipp, Craig, Becker, & Horstmann, 2013). These studies used a visual search paradigm (the “face-in-the-crowd paradigm”, FitC), in which participants have to decide as quickly and accurately as possible whether an array of faces (all displaying the same expression, e.g. all neutral/happy/angry) contains a deviating target face. In this paradigm, the happiness superiority effect manifested itself in faster reaction times and lower error rates for targets with happy expressions compared to angry but also other negative expressions (Becker et al., 2011; Savage et al., 2013). In emotion categorization tasks, a faster and more correct categorization of happy facial expressions was also observed (Leppänen & Hietanen, 2004; Leppänen, Tenhunen, & Hietanen, 2003).

Previous empirical studies suggest that there are several facial features that can influence the size of the happiness superiority effect, including sex and race (e.g., Becker, Kenrick, Neuberg, Blackwell, & Smith, 2007; Craig & Lipp, 2017; Bijlstra, Holland, & Wigboldus, 2010). Specifically, the happiness superiority effect was larger for female targets (Becker et al., 2007; Bucher & Voss, 2019; Bucher, Voss, Spaniol, Hische, & Sauer, 2019; Craig & Lipp, 2017; Hugenberg & Sczesny, 2006) and for male own-race targets compared to male other-race targets (Bijlstra et al., 2010; Craig, Mallan, & Lipp, 2012). In addition to this research, a recent study also found that facial *attractiveness* moderates the happiness superiority effect (Lindeberg, Craig, & Lipp, 2019). In four experiments, the authors found evidence for a faster and more accurate identification of happiness (versus anger) in attractive faces compared to unattractive faces, when presenting one target in each trial.

The evidence of a faster and more accurate identification of attractive faces displaying happiness can be explained through an interplay of two theoretical accounts: the attractiveness stereotype and the evaluative congruence account. The *attractiveness stereotype*, also known as the “what is beautiful is good” effect (Dion, Berscheid, & Walster, 1972), describes the phenomenon that humans believe physical attractiveness to be associated with a diverse range of positive attributes (for a meta-analysis, see Eagly, Ashmore, Makhijani, & Longo, 1991a, b). For example, attractiveness increases the likelihood of getting a job interview (Watkins & Johnston, 2000), entails higher ratings on favorable personality traits such as Agreeableness (Borkenau & Liebler, 1992, 1995; Smits & Cherhoniak, 1976), and higher intelligence ratings (Jackson, Hunter, & Hodge, 1995). Thus, the attractiveness stereotype suggests that attractiveness and positive attributes are strongly associated in the minds of people. To explain, why the pairing of attractive happy faces entails a faster and more accurate processing, one can additionally draw on the *evaluative congruence account* (Hugenberg, 2005; Hugenberg & Sczesny, 2006). The evaluative congruence account postulates that emotions are processed faster when they match the evaluation of a social cue that they are presented in conjunction with, for instance attractiveness. Based on the attractiveness stereotype account, we know that attractiveness is evaluated positively, as is a happy affect. Therefore, happiness and attractiveness are both positive cues and, based on the evaluative congruence account, should be processed faster than if the pairing of affect and a social cue were incongruent (e.g. angry + attractive).

However, the attractiveness stereotype also has a flipside: Humans also tend to ascribe *negative* qualities to *un*attractive persons (Dion et al., 1972). Based on the evaluative congruence account, one would therefore expect that unattractive faces paired with a negative emotion (e.g. anger) are also processed faster and more accurately. However, this effect was not observed in the one previous study that investigated the categorization of attractive and unattractive faces displaying happy and angry affect (Lindeberg et al., 2019). It is possible that the categorization task applied in this study was too easy and incurred very low error rates that might impede the detection of response biases. At the same time, the absence of a detection advantage for unattractive angry faces can also be seen as evidence against evaluative congruence. The present study aimed to replicate findings by Lindeberg and colleagues in a novel paradigm that overcomes some of the limitations of single target paradigms and includes eye-tracking analyses to assess underlying processes. This way, we hoped to shed light on whether unattractive-angry faces indeed do not entail a detection advantage (as suggested by the previous evidence) and which would reject the evaluative congruence account, or whether this effect would be present in a paradigm that leaves more room for variance in participant performance.

Specifically, the present study relied on a novel paradigm that incurs higher cognitive demands and lower accuracy rates than single-target tasks. The current paradigm might thus leave more room for motives to bias emotion perception and enables testing the evaluative congruence account for both attractive and unattractive faces. In the “mood-of-the-crowd paradigm” (MoC), participants have to judge the *overall* mood of the crowd by indicating whether more angry or happy faces are present in an array of faces (Bucher & Voss, 2019). Previous studies were able to replicate the happiness superiority effect in this setting: happy crowds were identified faster and more accurately than angry crowds (Bucher & Voss, 2019; Elias, Dyer, & Sweeny, 2017). The MoC paradigm has a number of advantages over the FitC paradigm. First, judging the mood of a crowd represents a more ecologically valid task than detecting a single face in a crowd, because crowds with multiple target emotions are highly prevalent in daily life (e.g., in class, at a sporting event, etc.). Moreover, the presentation of more than one emotional target prevents confusion about whether an observed effect results from a target or a crowd effect (see Bucher & Voss, 2019). As outlined above, the MoC task is also a more complex and cognitively demanding task and therefore does not incur similar floor effects for error rates compared to single target paradigms. Furthermore, the MoC task can be well combined with process tracing methods such as eye-tracking. Eye-tracking is less applicable to the FitC paradigm, for example, because the search process ends abruptly as soon as the participant detects the target, which makes it difficult to identify underlying search patterns.

The implementation of eye-tracking analysis is advantageous for the current investigation of the evaluative congruence account for facial attractiveness because it makes it possible to pinpoint the locus of the effect of attractiveness on emotion perception. If an attractiveness effect was apparent in eye movements (e.g., an increased probability and longer duration of fixations on happy-attractive/angry-unattractive faces), this would indicate that congruent stimuli (e.g. happy-attractive faces) are preferentially or selectively attended to and thus attentional processes guide the judgement of whether the crowd is perceived as predominantly happy or angry. In contrast, if participants showed no differences in their fixation patterns between congruent (happy-attractive/ angry-unattractive) and incongruent (happy-unattractive/angry-attractive) stimuli, but did show differences at the choice level, this would suggest effects in the evaluation phase (e.g., a biased response towards happiness for attractive crowds). In this case, participants attend to both congruent and incongruent stimuli in a similar way but the final judgment is biased towards one emotion, indicating that the evaluation of the selected faces influences the final judgment about the general mood of the crowd. Without the inclusion of eye-tracking measures, this differentiation is not possible.

## The current study

The aim of the current study was to test the evaluative congruence account, thus also aiming for conceptual replication of the findings by Lindeberg et al. (2019) in a new paradigm. The present study used the MoC paradigm, in which the participant’s task is to decide as fast and accurately as possible whether more angry or more happy faces are presented in a crowd of faces. The merits of this task (compared to single target paradigms) include increased cognitive demand (and less likelihood of floor effects), increased ecological validity, separation of crowd and target effects, and the possibility of including eye-tracking indices in addition to response times and accuracy rates. Note that in other paradigms (e.g., categorization task or FitC paradigm) the use of single targets prevents investigation of attentional preferences regarding specific emotions or attractiveness-emotion combinations. For example, in the FitC paradigm, the search process stops abruptly as soon as the target is detected and in single face categorization tasks, there is no possibility to investigate search asymmetries. The MoC paradigm, however, allows for analyzing such gaze patterns. An inclusion of eye-tracking was desirable in this case to test whether perceptual preferences guide the evaluation of the crowd, indicated by an increased fixation rate on happy attractive and angry unattractive faces, or whether attractiveness influences the speed and accuracy of the judgements itself, indicated by a lack of an attractiveness effect in gaze movements. Lastly, the present study aimed to target a confound that was present in the studies by Lindeberg et al. (2019), including the fact that attractive female faces were consistently rated as more attractive compared to attractive male faces, whereas there was no difference for unattractive male and female faces. Thus, we used the same selection criterion for target faces as Lindeberg et al. (2019) in our first experiment, but matched target gender and attractiveness in the second experiment. Using these different selection criteria, we were able to test whether the gender-attractiveness imbalance accounts for the reported attractiveness effect on emotion perception.

We derived our hypotheses based on the evaluative congruence account (Hugenberg, 2005; Hugenberg & Sczesny, 2006) which suggests a processing advantage for emotions that are evaluated congruently as the social cue they are paired with (herein attractiveness). Therefore, we hypothesized that participants would identify attractive crowds more often as happy than unattractive crowds. Reversely, we also expected that participants would identify unattractive crowds more often as angry than attractive crowds. Moreover, we hypothesized that attractive crowds containing a higher number of happy faces, and unattractive crowds containing a higher number of angry expressions, should entail faster and more accurate responses. This effect has previously been supported by Lindeberg et al. (2019), who observed a faster and more accurate categorization of happy attractive compared to angry attractive expressions. Further corroborating this, there is evidence that participants evaluate happy faces as more attractive than faces displaying negative emotions (Mueser, Grau, Sussman, & Rosen, 1984). Moreover, Golle, Mast and Lobmaier (2014) showed that judgment of relatively happier compared to neutral faces is facilitated when those faces were attractive. Based on evaluative congruence, we expected a general tendency to judge attractive crowds as being happy and unattractive crowds as being angry more frequently. With regard to eye-tracking indices, we expected higher fixation rates and fixation durations on happy-attractive and angry-unattractive faces than on happy-unattractive or angry-attractive faces, based on a higher salience of congruently evaluated social cues.

## Experiment 1

### Method

#### Participants

Prior to recruiting participants, we conducted a power analysis using G*Power 3 (Faul, Erdfelder, Lang, & Buchner, 2007) to determine the required sample size. The required sample size to detect an effect of medium size^1^ with a power of 0.80 and an alpha-error of 0.05 in a repeated measures ANOVA setting was 34. Thirty-four adults participated in the study (*M*_age_ = 23.06 years, *SD* = 5.22, range 19–47; 50% female). We recruited participants from a large participant pool at a German university, in which students of diverse subjects are registered. Before participants took part in the study, they received information about the study details and provided written, informed consent. As compensation for the study, participants could choose between course credit or a financial compensation of five Euros.

#### Stimulus material

Following Lindeberg et al. (2019), we chose the stimulus material for the first experiment by selecting the most and least attractive faces from a pool of faces. We selected the stimulus faces from the Chicago Face Database (Ma, Correll, & Wittenbrink, 2015), restricting the stimulus material to faces of Caucasian individuals that provided both angry and happy (closed mouth) expressions. We used the attractiveness ratings of the remaining 37 female and 36 male faces provided by the norming data of the Chicago Face Database and selected nine faces that were rated the most attractive and nine faces that achieved the lowest attractiveness ratings for female and male faces, respectively. This procedure resulted in 18 female (nine attractive and nine unattractive) and 18 male (nine attractive and nine unattractive) individuals with happy and angry expressions available. The attractiveness ratings of the ‘attractive’ female models (*M* = 4.60, *SD* = 0.32; Models 3, 11, 12, 15, 22, 24, 25, 27 and 29) differed from those of the ‘unattractive’ female models (*M* = 2.32, *SD* = 0.45; Models 2, 8, 10, 23, 26, 28, 30, 34 and 37). Likewise, the attractiveness of the ‘attractive’ male models (*M* = 3.89, *SD* = 0.46; Models 3, 4, 6, 9, 14, 15, 24, 29 and 33) differed from that of the ‘unattractive’ male models (*M* = 2.36*, SD* = 0.16; Models 2, 10, 17, 19, 35, 37, 38, 39, 41).^2^ Lighting and visual contrast were similar across all faces in the set.

#### Procedure

The experiment was run on a Dell laptop computer. Stimulus displays and response time measurement were controlled by a C program using SDL libraries (www.libsdl.org). We used a SMI RED250MOBILE eye-tracker to record gaze movements at a frequency of 250 Hz. We defined fixations using the criteria of a minimal duration of 100 ms and maximal dispersion of 100 pixels.

After providing written, informed consent, participants were seated approximately 60 cm in front of the laptop computer. First, participants provided demographic information and then received information that the upcoming task was to judge whether the crowd contained more happy or angry faces. We specifically instructed participants to make their decisions as fast and accurately as possible to prevent them from simply counting the presented faces. The experiment comprised a practice block of 16 trials and two experimental blocks of 50 trials each. Prior to each block (practice block and experimental blocks) we used a 9-point-calibration for calibrating the eye-tracker. Each calibration was followed by a 4-point validation. After a successful validation (participants’ gaze was within 1° visual angle), participants started with the experimental trials. In case of a non-successful validation, we repeated calibration and validation.

In each trial, participants saw a crowd of 18 faces (each 1.4 cm × 1.9 cm). Faces were randomly allocated on the screen in front of a light-grey background (34.5 cm × 19.5 cm) to ensure naturalistic eye movements, which is not possible when presenting faces in a circle or matrix (due to systematic scanning paths: e.g. clockwise or row-wise). To avoid overlap between the pictures, we ensured a minimum distance between the edges of the pictures (minimum distance was 3.4 cm). Crowds contained a varying number of angry faces (6, 8, 10 and 12 angry faces),^3^ and crowds were either completely made up of attractive or unattractive faces. The different compositions of angry and happy faces within the crowds (six angry/12 happy, eight angry/ten happy, ten angry/eight happy, 12 angry/six happy*)* occurred equally often in attractive and unattractive crowds to prevent any systematic confound. Moreover, crowds always contained the same number of female and male faces (e.g., in case of ten angry and eight happy faces the crowd contained five female angry and five male angry expressions as well as four female happy and four male happy expressions). All presented face pictures within one trial were from different individuals.

Each block started with two warm-up trials. The eight trial combinations (attractiveness of presented faces × number of angry pictures) were presented six times in random order in each experimental block. Prior to each trial, a fixation cross was presented at the center of the screen before the crowd was shown. Only when participants fixated on this cross, the crowd appeared. If participants did not focus on the fixation cross until 5000 ms were exceeded, a recalibration started. The crowd remained visible until participants gave their response. If participants’ responses were slower than 8000 ms, a message (“please try to respond faster”) appeared. Participants could press the S-key and the K-key to indicate whether they perceived the crowd as predominantly happy or angry. The assignment of the emotions to the keys was balanced across participants. As a reminder, the words “happy” and “angry” appeared on the respective sides on the bottom of the screen. Subsequent to the eye-tracking experiment, participants rated the happy and angry faces on attractiveness, happiness, and anger on a 7-point Likert scale in a randomized sequence.

#### Analysis

A 2 (attractiveness: attractive, unattractive) × 2 (trial type: mainly happy, mainly angry^4^) repeated measures ANOVA was conducted to analyze responses, accuracy rates and response times. For the analyses of the number of fixations and fixation duration on the respective faces, the emotion of the focused picture (happy vs. angry) served as an additional within-subjects factor. This way, we assessed how many faces of each type received at least one fixation during a trial and how long each type of face was fixated. We excluded responses faster than 500 ms and slower than 8000 ms from the response time analysis (0.51% of all trials). Additionally, we adjusted for participants’ gender as a covariate in all analyses. If not reported in the text, gender did not show any significant main effects or interactions.

## Results

### Manipulation check

Attractiveness ratings were obtained in a 2 (emotional expression: happy, angry) × 2 (attractiveness: attractive, unattractive) × 2 (target gender: female, male) repeated measures ANOVA. There was a main effect of attractiveness, *F*(1, 32) = 472.53, *p* < 0.001, *η*^2^_p_ = 0.94, 95% CI [0.89, 0.95], emotional expression, *F*(1, 32) = 15.53, *p* < 0.001, *η*^2^_p_ = 0.33, 95% CI [0.11, 0.49], and gender, *F*(1, 32) = 46.78, *p* < 0.001, *η*^2^_p_ = 0.59, 95% CI [0.39, 0.70], confirming that attractive, happy and female targets were rated as being more attractive compared to the unattractive, angry and male targets. Moreover, we found a significant interaction between attractiveness and target gender, *F*(1, 32) = 97.83, *p* < 0.001, *η*^2^_p_ = 0.75, 95% CI [0.61, 0.82]. Follow-up pairwise comparisons revealed that for attractive faces, female targets were perceived as more attractive compared to male targets, *t*(33) = 9.06, *p* < 0.001, *d* = 1.55, whereas the unattractive male and female targets did not differ significantly, *t* < 1.

We ran the same analysis for the emotional intensity ratings. There was a significant main effect of attractiveness, *F*(1, 32) = 32.24, *p* < 0.001, *η*^2^_p_ = 0.50, 95% CI [0.28, 0.63], and gender, *F*(1, 32) = 40.48, *p* < 0.001, *η*^2^_p_ = 0.56, 95% CI [0.34, 0.68], showing that attractive and female targets were perceived as showing a higher emotional intensity. The main effects were qualified by an attractiveness × target gender interaction, *F*(1, 32) = 15.89, *p* < 0.001, *η*^2^_p_ = 0.33, 95% CI [0.12, 0.50] and an attractiveness × emotional expression interaction, *F*(1, 32) = 71.26, *p* < 0.001, *η*^2^_p_ = 0.69, 95% CI [0.52, 0.77]. Follow-up pairwise comparisons showed that female expressions were rated as more emotionally intensive compared to male faces both for attractive, *t*(33) = 2.37, *p* = 0.024, *d* = 0.41, and unattractive targets, *t*(33) = 7.45, *p* < 0.001, *d* = 1.28. However, this difference was much more pronounced for unattractive faces, *t*(33) = 3.96, *p* < 0.001, *d* = 0.68. Additionally, pairwise comparisons revealed that happiness was expressed more strongly on attractive compared to unattractive faces, *t*(33) = 8.37, *p* < 0.001, *d* = 1.44, whereas there was no significant difference for angry faces, *t*(33) = − 1.90, *p* = 0.066, *d* = 0.33.

Mean and standard deviations of the attractiveness and emotional intensity ratings are summarized in Table 1.

**Table 1:** Attractiveness and emotional intensity ratings for happy and angry female and male faces from both experiments

Measures	Female		Male	
	Happy	Angry	Happy	Angry
Experiment 1				
Attractiveness				
Attractive	5.63 (0.69)	5.06 (0.86)	3.99 (1.23)	3.52 (1.04)
Unattractive	2.76 (1.07)	2.13 (0.84)	2.66 (1.13)	2.11 (1.04)
Emotional intensity				
Attractive	5.59 (0.73)	5.41 (0.73)	5.36 (0.55)	5.35 (0.63)
Unattractive	5.20 (0.66)	5.69 (0.64)	4.76 (0.73)	5.23 (0.71)
Experiment 2				
Attractiveness				
Attractive	4.55 (0.76)	3.70 (0.96)	4.17 (0.94)	3.52 (1.10)
Unattractive	3.40 (0.84)	2.51 (0.82)	3.13 (0.84)	2.36 (0.75)
Emotional intensity				
Attractive	5.18 (0.69)	5.19 (0.74)	5.11 (0.71)	4.97 (0.88)
Unattractive	4.92 (0.69)	5.42 (0.80)	5.08 (0.71)	5.23 (0.75)

Values in parantheses represent 1 *SD*

### Response time

**Fig. 1:**
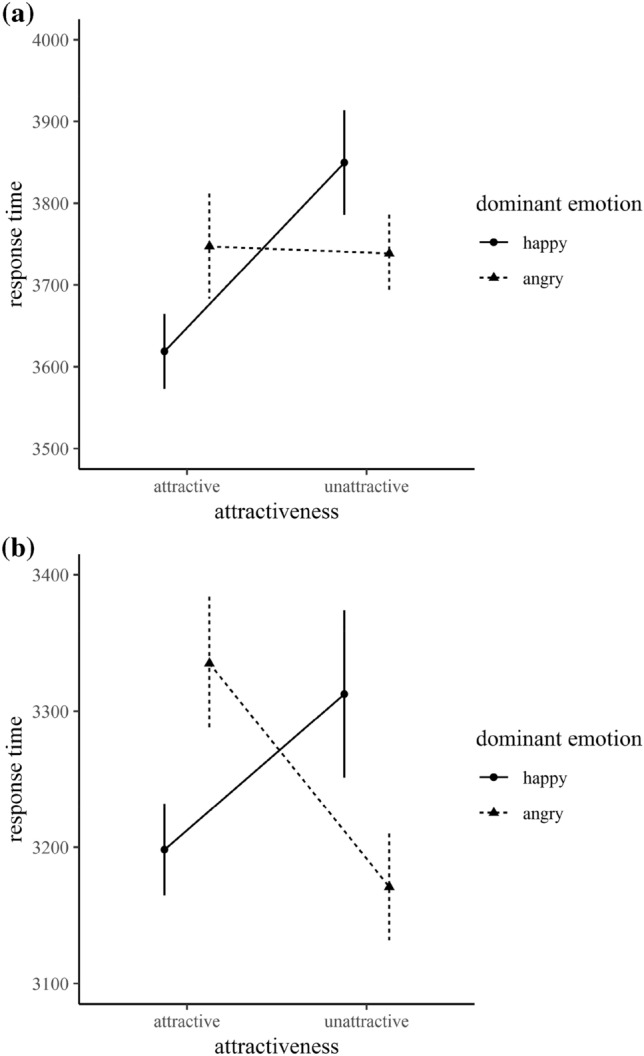
Interaction effect of dominant emotion and attractiveness on response times (in ms) for experiment 1 (**a**) and experiment 2 (**b**). Trials with ten and 12 happy expressions were combined as well as trials with ten and 12 angry expressions. Error bars indicate standard errors

We computed mean correct response times for each participant and each factorial combination. There was a significant interaction between attractiveness and trial type, *F*(1, 32) = 6.18, *p* = 0.018, *η*^2^_p_ = 0.16, 95% CI [0.02, 0.34]. Follow-up pairwise comparisons revealed that participants were faster when more happy faces were presented in attractive crowds compared to unattractive crowds, *t*(33) = 2.97, *p* = 0.006, *d* = 0.51, whereas there was no difference when more angry faces were shown, *t* < 1 (Fig. 1a).

### Response

**Fig. 2:**
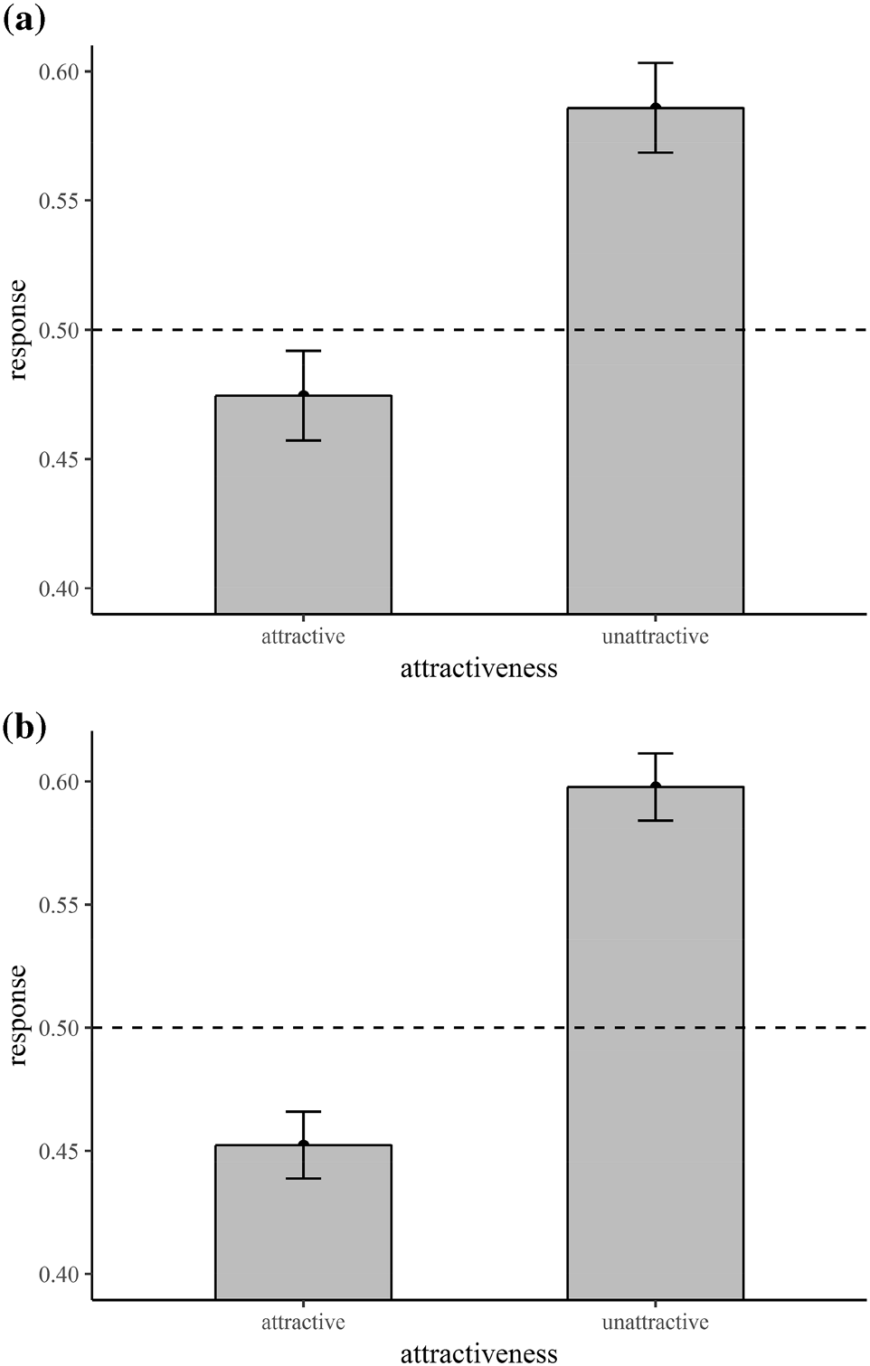
Main effect of attractiveness on response tendency (0 = happy, 1 = angry) for experiment 1 (**a**) and experiment 2 (**b**). Error bars indicate standard errors

Mean responses (proportion of “angry” responses) were calculated for each participant and each factorial combination. A main effect of attractiveness, *F*(1, 32) = 32.53, *p* < 0.001, *η*^2^_p_ = 0.50, 95% CI [0.28, 0.63], and a main effect of trial type emerged, *F*(1, 32) = 315.00, *p* < 0.001, *η*^2^_p_ = 0.91, 95% CI [0.85, 0.93]. Attractive crowds were judged as “happy” and unattractive ones as “angry” more frequently (Fig. 2a). Participants evaluated crowds containing more happy faces as being happy more often compared to those with more angry faces.

### Accuracy

**Fig. 3:**
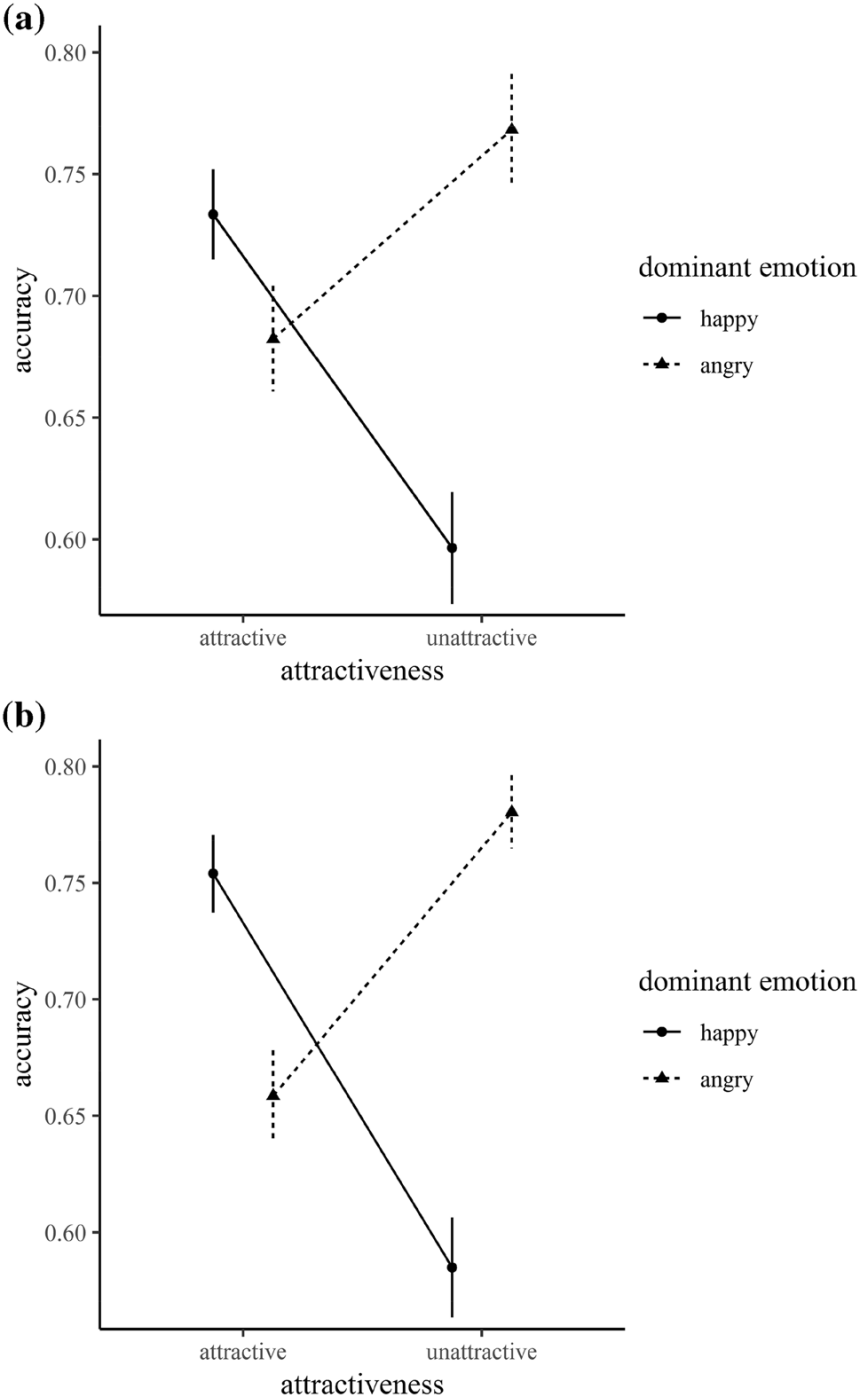
Interaction effect of dominant emotion and attractiveness on accuracy (0 = false, 1 = correct) for experiment 1 (**a**) and experiment 2 (**b**). Trials with ten and 12 happy expressions were combined as well as trials with ten and 12 angry expressions. Error bars indicate standard errors

Mean accuracies were calculated for each participant and each factorial combination. There was a main effect of trial type, *F*(1, 32) = 4.23, *p* = 0.048, *η*^2^_p_ = 0.12, 95% CI [0.00, 0.29], indicating slightly increased accuracy rates in trials with more angry faces. This main effect was qualified by an attractiveness × trial type interaction, *F*(1, 32) = 32.53, *p* < 0.001, *η*^2^_p_ = 0.50, 95% CI [0.28, 0.63]. Follow-up *t* tests indicated that when more happy faces appeared in a crowd, participants showed higher accuracy rates for attractive crowds, *t*(33) = 6.31, *p* < 0.001, *d* = 1.08, and the reversed pattern when confronted with unattractive faces, *t*(33) = − 3.34, *p* = 0.002, *d* = 0.57 (Fig. 3a).

### Fixation duration

Mean durations were calculated for each participant and each factorial combination. There was no significant main or interaction effect of any of the investigated factors, *F* < 2.10, *p* > 0.16, *η*^2^_p_ < 0.06.

### Number of fixations

**Fig. 4:**
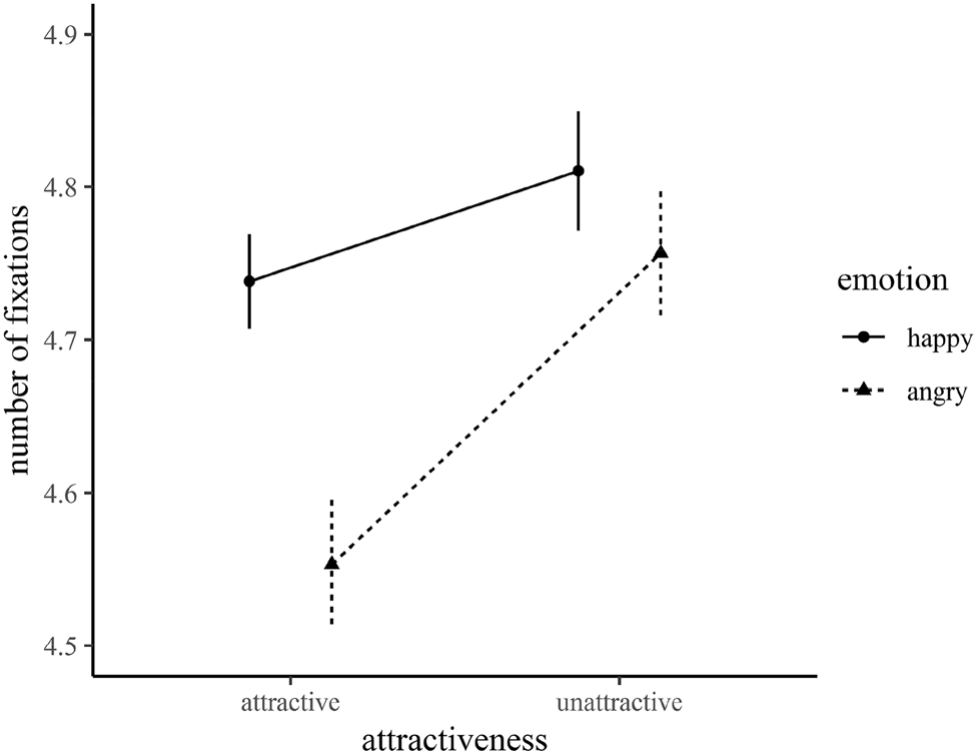
Interaction effect of target emotion and attractiveness on number of fixations. Error bars indicate standard errors

The number of fixations assesses how many faces of each type received at least one fixation during a trial. There was a main effect of emotional expression, *F*(1, 32) = 8.30, *p* = 0.007, *η*^2^_p_ = 0.21, 95% CI [0.04, 0.38], and attractiveness, *F*(1, 32) = 10.20, *p* = 0.003, *η*^2^_p_ = 0.24, 95% CI [0.05, 0.42], indicating that happy faces and unattractive faces were fixated more frequently compared to angry and attractive faces. Moreover, we found a significant interaction between attractiveness and emotional expression, *F*(1, 32) = 4.65, *p* = 0.039, *η*^2^_p_ = 0.13, 95% CI [0.00, 0.30]. Follow-up pairwise comparisons revealed that in unattractive crowds happy and angry expressions were fixated to the same degree, *t* < 1, whereas in attractive crowds significantly more happy faces were fixated, *t*(33) = 3.85, *p* = 0.001, *d* = 0.66 (Fig. 4). There was a significant interaction between emotional expression and trial type, *F*(1, 32) = 301.35, *p* < 0.001, *η*^2^_p_ = 0.90, 95% CI [0.84, 0.39], with more fixations on angry (happy) faces when more angry (happy) faces were presented. Lastly, an interaction between all factors emerged,^5^*F*(1, 32) = 4.55, *p* = 0.041, *η*^2^_p_ = 0.13, 95% CI [0.00, 0.30].^6^

## Discussion

In experiment 1, we assessed whether stimulus attractiveness has an effect on the happiness superiority effect in visual search, specifically in the MoC paradigm. We hypothesized a faster and more accurate perception of attractive crowds containing more happy faces and of unattractive crowds comprising more angry faces. We expected a response bias towards happiness in attractive crowds and towards anger in unattractive crowds. Results revealed that participants judged crowds containing attractive faces as happy more often than crowds containing unattractive faces. Moreover, participants were faster and more accurate when crowds were both attractive and containing many happy faces. In line with the evaluative congruence account, we also found that participants judged unattractive crowds as angry more frequently, and that judgments were more accurate in trials with more angry targets presented. This is a novel finding, as the previous study testing this model (Lindeberg et al., 2019) only found support for part of the evaluative congruence account, showing an advantage for attractive-happy faces but not for unattractive-angry targets. Further extending previous findings, we included eye-tracking analyses that revealed that fixations occurred more frequently on happy compared to angry facial expressions when attractive faces were presented.

The results supported our hypotheses and showed medium to large effect sizes, but were limited by an imbalance between target gender and attractiveness. Thus, we cannot completely rule out the possibility that this gender-attractiveness disparity affected our results. Mirroring the findings by Lindeberg et al. (2019), the difference between attractiveness ratings was also larger for attractive female compared to attractive male faces. Furthermore, we found that participants rated happy attractive faces as more emotionally intense than happy unattractive faces. Although we believe that attractiveness influenced the emotion ratings to the same degree as accuracy, response tendency and response times in the MoC task, other explanations might be possible as well. It is imaginable that the completion of the MoC task influenced the following ratings, because we observed the same pattern of results for the experiment and the ratings. Moreover, it is possible that unattractive targets showed less intense happy expressions than attractive targets. To address these issues, we conducted a second experiment and chose different selection criteria for the stimulus material.

## Experiment 2

In experiment 1, the focus when choosing the stimulus material was to achieve the greatest possible attractiveness difference between unattractive and attractive targets. This approach leads to a larger difference in perceived attractiveness and maximizes the chances of finding an attractiveness effect. Lindeberg and colleagues (2019) critically discussed this selection procedure and demonstrated that it leads to a greater difference in rated attractiveness for attractive female compared to attractive male targets. As target gender also influences emotion perception to a large degree, it might be the case that results from experiments using this selection method are somewhat confounded. Therefore, in experiment 2, we used different selection criteria for the material to ensure that attractive female and male targets as well as unattractive male and female targets match with regard to their perceived attractiveness. Furthermore, we used the emotion intensity ratings for the neutral faces provided in the norming data of the Chicago Face Database to better match the attractive and unattractive face pictures.^7^ Despite these modifications, the experimental setting and hypotheses in experiment 2 were identical to experiment 1.

## Methods

### Participants

Based on the same power analysis as in experiment 1,^8^ we recruited 34 adults (*M*_age_ = 23.26 years, *SD* = 5.66, range 18–46; 53% female) via a large participant pool of a German university. Before starting with the experiment, participants received information about the upcoming task and provided written, informed consent. Again, participants could receive course credit or five Euros as compensation for their participation.

### Stimulus material

We again selected the stimulus material from the Chicago Face Database (Ma et al., 2015), considering only pictures of Caucasian men and women that provided both happy and angry expressions. In addition to the attractiveness ratings, happiness and anger ratings were taken into account when choosing the stimulus material. Happiness and anger ratings of the neutral face pictures of the unattractive and attractive male and female pictures were matched to control for possible emotional intensity differences between the pictures. Furthermore, we matched target gender and perceived attractiveness, so that attractive males and females as well as unattractive males and females had similar attractiveness rating. We again selected 18 female (nine attractive and nine unattractive) and 18 male (nine attractive and nine unattractive) individuals. When comparing the attractiveness ratings, the attractive female (*M*_att_ = 3.95, *SD*_att_ = 0.38; *M*_happy_ = 2.47, *SD*_happy_ = 0.49; *M*_angry_ = 2.60, *SD*_angry_ = 0.65; Models 6, 11, 13, 15, 16, 18, 21, 25 and 31) and unattractive female models (*M*_att_ = 2.79, *SD*_att_ = 0.17; *M*_happy_ = 2.41, *SD*_happy_ = 0.54; *M*_angry_ = 2.46, *SD*_angry_ = 0.76; Models 5, 7, 8, 19, 23, 28, 30, 36 and 37) as well as the attractive male (*M*_att_ = 3.89, *SD*_att_ = 0.46; *M*_happy_ = 2.63, *SD*_happy_ = 0.32; *M*_angry_ = 2.21, *SD*_angry_ = 0.39; Models 3, 4, 6, 9, 14, 15, 24, 29 and 33) and unattractive male models (*M*_att_ = 2.71*,* *SD*_att_ = 0.13; *M*_happy_ = 2.48, *SD*_happy_ = 0.67; *M*_angry_ = 2.39, *SD*_angry_ = 0.50; Models 12, 13, 20, 21, 23, 25, 32, 34 and 37) differed in rated attractiveness, respectively, but not with regard to happiness or anger. Lighting and visual contrast were similar across all faces in the set.

As the attractiveness, happiness and anger ratings were only available for the neutral face expressions, the happy and angry expressions of the attractive and unattractive male as well as female individuals were additionally rated by 45 participants (*M*_age_ = 34.98 years, *SD* = 13.60, range 19–59; 73% female).^9^ We present the results for the attractiveness and emotional intensity ratings in the results section.

### Procedure

The experimental setup and procedure were identical to those in experiment 1. The only difference was with regard to the stimulus material and that the emotional face expressions were rated prior to the study regarding attractiveness and emotional intensity (happiness, anger) by an independent sample to prevent transfer effects.

### Analysis

The analytic strategy was identical to that in experiment 1. Again, we excluded responses faster than 500 ms and slower than 8000 ms from the response time analysis (0.43% of all trials).

## Results

### Manipulation check

Ratings from the 45 participants of the pretest were used to analyze perceived attractiveness and emotional intensity. There were main effects of attractiveness, *F*(1, 43) = 169.28, *p* < 0.001, *η*^2^_p_ = 0.80, 95% CI [0.70, 0.85], target emotion, *F*(1, 43) = 40.79, *p* < 0.001, *η*^2^_p_ = 0.49, 95% CI [0.30, 0.61], and target gender, *F*(1, 43) = 14.15, *p* = 0.001, *η*^2^_p_ = 0.25, 95% CI [0.08, 0.40], indicating that attractive, happy and female faces were rated as more attractive compared to unattractive, angry and male faces. This time, there was no significant interaction between attractiveness and target gender, *F*(1, 43) = 0.63, *p* = 0.432, *η*^2^_p_ = 0.01, 95% CI [0.00, 0.12]. Lastly, we found a significant interaction between target emotion and target gender, *F*(1, 43) = 9.56, *p* = 0.003, *η*^2^_p_ = 0.18, 95% CI [0.04, 0.34]. Follow-up pairwise comparisons revealed that happy faces were rated more attractively compared to angry faces both for female, *t*(44) = 8.25, *p* < 0.001, *d* = 1.23, and male faces, *t*(44) = 6.74, *p* < 0.001, *d* = 1.00, however, this difference was significantly larger for female faces, *t*(44) = 2.76, *p* = 0.008, *d* = 0.41.

With regard to emotional intensity, there was a significant main effect of target gender, *F*(1, 43) = 13.48, *p* = 0.001, *η*^2^_p_ = 0.24, 95% CI [0.07, 0.39], and participants’ gender, *F*(1, 43) = 9.64, *p* = 0.003, *η*^2^_p_ = 0.18, 95% CI [0.02, 0.37], indicating that female faces were perceived as more emotionally intense and that female raters gave higher intensity ratings. The main effect of target gender was qualified by an interaction between target gender and target emotion, *F*(1, 43) = 8.13, *p* = 0.007, *η*^2^_p_ = 0.16, 95% CI [0.03, 0.32]. Whereas happy and angry faces were perceived as equally intense in male faces, *t* < 1, angry female faces were rated more intensely compared to happy female faces, *t*(44) = − 2.65, *p* = 0.011, *d* = 0.40. Furthermore, there was a significant interaction between attractiveness and target emotion, *F*(1, 43) = 34.89, *p* < 0.001, *η*^2^_p_ = 0.45, 95% CI [0.26, 0.58]. Happy attractive faces were rated more intensely than happy unattractive faces, *t*(44) = 3.76, *p* < 0.001, *d* = 0.56, whereas the reverse pattern was found for angry expressions, *t*(44) = − 4.94, *p* < 0.001, *d* = 0.74. Lastly, a significant interaction between participants’ gender and target gender emerged, *F*(1, 43) = 11.34, *p* = 0.001, *η*^2^_p_ = 0.21, 95% CI [0.05, 0.37]. Male participants rated female faces more emotionally intense compared to male face, *t*(44) = 4.56, *p* = 0.001, *d* = 0.68, whereas there was no such difference for female participants, *t* < 1.^10^

Mean and standard deviations of the attractiveness and emotional intensity ratings are summarized in Table 1.

### Response time

We computed mean correct response times for each participant for each factorial combination. Again, there was a significant interaction between attractiveness and trial type, *F*(1, 32) = 10.39, *p* = 0.003, *η*^2^_p_ = 0.25, 95% CI [0.06, 0.42]. Follow-up pairwise comparisons showed that in attractive crowds participants were faster when crowds contained more happy compared to angry faces, *t*(33) = 2.57, *p* = 0.015, *d* = 0.44, whereas in unattractive crowds the pattern pointed towards the opposite direction, *t*(33) = − 1.91, *p* = 0.065, *d* = 0.33 (Fig. 1b). Lastly, there was a significant effect of participants’ gender, *F*(1, 32) = 4.55, *p* = 0.041, *η*^2^_p_ = 0.12, 95% CI [0.00, 0.34], indicating that women were faster in judging the mood of the crowd.

### Response

We calculated mean responses (proportion of “angry” responses) for each participant and each factorial combination. Again, there was a main effect of attractiveness, *F*(1, 32) = 68.62, *p* < 0.001, *η*^2^_p_ = 0.68, 95% CI [0.50, 0.77], and of trial type, *F*(1, 32) = 316.51, *p* < 0.001, *η*^2^_p_ = 0.91, 95% CI [0.85, 0.93]. Participants tended to judge attractive crowds more often as happy, whereas they judged unattractive crowds more often as angry (Fig. 2b). Participants evaluated crowds dominated by happy faces as being happy more often compared to those containing more angry expressions.

### Accuracy

Mean accuracies were calculated for each participant and each factorial combination. A main effect of trial type reached significance, *F*(1, 32) = 5.37, *p* = 0.027, *η*^2^_p_ = 0.14, 95% CI [0.01, 0.32], showing higher accuracy rates in trials with more angry expressions. This main effect was qualified by an attractiveness × trial type interaction, *F*(1, 32) = 68.62, *p* < 0.001, *η*^2^_p_ = 0.68, 95% CI [0.50, 0.77]. Follow-up *t* tests revealed that accuracy rates were increased when more happy faces were presented in attractive compared to unattractive crowds, *t*(33) = 6.50, *p* < 0.001, *d* = 1.11, and the reversed pattern was found for angry expressions, *t*(33) = − 5.41, *p* < 0.001, *d* = 0.93 (Fig. 3b).

### Fixation duration

Mean durations were calculated for each participant and each factorial combination. Male participants fixated significantly longer on the presented faces compared to female participants, *F*(1, 32) = 4.92, *p* = 0.034, *η*^2^_p_ = 0.13, 95% CI [0.01, 0.31]. The only other significant effect was a three-way interaction between emotional expression, trial type and participants’ gender, *F*(1, 32) = 4.47, *p* = 0.042, *η*^2^_p_ = 0.12, 95% CI [0.00, 0.30]. Whereas there was no significant interaction between emotional expression and trial type for male participants, *F*(1, 15) = 1.14, *p* = 0.303, *η*^2^_p_ = 0.07, 95% CI [0.00, 0.30], there was a marginal significant interaction for female participants, *F*(1, 17) = 3.82, *p* = 0.067, *η*^2^_p_ = 0.18, 95% CI [0.00, 0.41], indicating that women fixated longer on angry (happy) faces when the crowds consisted of mainly angry (happy) faces.

### Number of fixations

This measure indicates how many faces of each type received at least one fixation during a trial. A significant interaction between emotional expression and trial type was found, *F*(1, 32) = 201.13, *p* < 0.001, *η*^2^_p_ = 0.86, 95% CI [0.77, 0.90], with more fixations on angry (happy) faces when more angry (happy) faces were presented. Lastly, there was a significant interaction between attractiveness and trial type, *F*(1, 32) = 9.31, *p* = 0.005, *η*^2^_p_ = 0.23, 95% CI [0.05, 0.40]. A higher number of faces was fixated in attractive compared to unattractive crowds when more angry faces were presented, *t*(33) = 2.38, *p* = 0.024, *d* = 0.41, whereas there was no significant difference in crowds containing more happy faces, *t*(33) = − 1.69, *p* = 0.101, *d* = 0.29.

## Discussion

In experiment 2, we replicated the main findings of experiment 1 in a different sample and using new stimulus material that was better matched for attractiveness between female and male stimuli. Again, participants evaluated attractive crowds as happy more frequently than unattractive crowds. Reversely, participants evaluated unattractive (compared to attractive) crowds more often as angry. Moreover, participants were faster and more accurate in judging attractive crowds dominated by happy faces and unattractive crowds dominated by angry expressions. These findings are in line with the evaluative congruence account, which suggests a facilitated perception when the presented emotion matches the evaluation of the respective social cue. Extending previous findings by Lindeberg et al. (2019), we found evidence supporting the evaluative congruence account not only for attractive, but also for unattractive faces.

In contrast to the findings for reaction times and choices, which were well in line with the findings from experiment 1, eye-tracking results did not replicate as closely. In experiment 1, we observed an increased number of fixations on happy attractive compared to angry attractive faces but this was not the case in experiment 2. It is possible that the difference in material explains this discrepancy. As described above, we performed a new matching of the target material to reduce attractiveness differences between female and male faces that were present in experiment 1. While successful in this regard, the new matching also resulted in smaller overall differences between attractive and unattractive targets. It is possible that the attention capturing power of the attractive smiling faces was thereby diminished, leading to a similar allocation of attention to attractive and unattractive emotional face expressions.

Even though we were able to match the perceived attractiveness of female and male targets in experiment 2, we again found that participants rated happy attractive faces as more emotionally intense compared to happy unattractive faces and the opposite for angry attractive and unattractive face pictures. This time, we collected ratings in a separate sample, ruling out the possibility that the completion of the MoC task influenced the consecutive rating. Furthermore, we consider it unlikely that, again, the emotional intensity of the happy (angry) unattractive faces was truly lower (higher) compared to the happy (angry) attractive faces as we used two different stimulus sets from the Chicago Face Database. Therefore, the most plausible explanation might be that attractiveness also influenced/biased the emotion intensity ratings. Using computer-generated emotional expressions matched for attractiveness, gender, and emotional intensity would be necessary in future investigations to ensure equal intensities of the emotional faces.

## General discussion

The present study set out to test the evaluative congruence account, which assumes a faster perception of an emotion when it matches the evaluation of a social cue, in two experiments. The social cue tested in the present study was attractiveness and we employed a visual search paradigm, the MoC task, in which participants’ task is to judge the overall mood of a crowd (instead of detecting single emotional targets as in previous studies, see Lindeberg et al., 2019). Based on the evaluative congruence account, we expected to see a faster and more accurate identification of attractive crowds dominated by happy faces and of unattractive crowds dominated by angry faces. Further extending previous studies, we also incorporated eye-tracking to assess potential mechanisms underlying evaluative congruence. For the eye-tracking indices, we expected higher fixation rates on happy faces in attractive crowds and higher fixation rates on angry faces in unattractive crowds.

Across both experiments, we found evidence for an influence of attractiveness on emotion perception. Participants in both samples evaluated attractive crowds of faces as happy more frequently. Moreover, their evaluations were faster and more accurate in attractive crowds dominated by happy faces. These findings are in line with the evaluative congruence account (Hugenberg, 2005; Hugenber & Sczesny, 2006). The results of the two studies also corroborate recent evidence by Lindeberg et al. (2019), who found that participants categorized attractive happy faces faster and more accurately than unattractive happy faces.

In contrast to previous studies, we also found evidence for evaluative congruence with regard to unattractive-angry faces. Participants rated unattractive crowds as angry more frequently and evaluated these crowds faster and more accurately, when they were dominated by angry faces. Therefore, we were not only able to find support for the evaluative congruence account with regard to attractive crowds of faces, but also for unattractive facial expressions. The reason for this might be that the MoC paradigm exerts higher cognitive demands and thus prevents floor effects (i.e. very low error rates with little variance) from occurring.

A further limitation that was present in previous studies we also observed in the present experiment 1. Participants rated attractive female faces as more attractive than attractive male faces, whereas there was no such difference with respect to unattractive male and female faces (and the same limitation afflicted studies by Lindeberg et al., 2019). To rule out the possibility that the happiness superiority effect for attractive crowds might be due to this dissimilarity, we matched female and male faces on attractiveness for the second experiment. Even though this led to smaller attractiveness differences between the unattractive and attractive faces, we were able to replicate our findings.

To additionally address potential mechanisms underlying the attractiveness effect on emotion perception, we combined the MoC paradigm with eye-tracking analyses. We hypothesized that evaluative congruence would manifest in higher fixation rates and longer fixation durations on happy-attractive and angry-unattractive faces than on happy-unattractive or angry-attractive faces. We partly found this only in the first experiment, where participants showed a higher number of fixations on happy compared to angry attractive faces. In the second experiment, we found no such evidence. One explanation might be the reduced attractiveness difference between the attractive and unattractive crowds as a result of the matching procedure in study 2. Moreover, it is necessary to differentiate that fixation durations on the happy and angry attractive faces were similar in study 1. In contrast, happy attractive faces were fixated more numerously compared to angry attractive faces in the first experiment. This indicates that attentional processes manifest in a biased selective attention towards happy attractive faces but not in a longer fixation duration for the happy attractive faces. However, compared to the large effects with regard to accuracy, response tendencies, and response times, only small effects on the eye-tracking variables were found in experiment 1. Therefore, we argue that differences in mood judgements in attractive compared to unattractive crowds are likely only slightly influenced by attentional processes but manifest more strongly in the evaluation phase.

A further, broader implication of the present studies is that it could be necessary to control for attractiveness when investigating the influence of other social cues (e.g. gender or race) on emotion perception, or even in visual tasks with emotional targets in general (Lindeberg et al., 2019). It is possible that past studies have confounded constructs such as target gender and attractiveness, and therefore the selection of the stimulus material may have exaggerated or underestimated effects of social cues on emotion perception (Lindeberg et al., 2019). Hence, controlling for attractiveness when selecting targets appears to be highly important. While knowledge about the far-reaching effects of other traits such as target gender or race are relatively well known and well addressed in the field, the same does currently not apply to attractiveness. Our findings thus also imply a need for existing and developing stimulus databases to obtain attractiveness ratings to allow researchers to account for this factor in their studies.

### Limitations

The present experiments entailed several limitations. First, we found that attractive happy and unattractive angry faces were rated as more emotionally intense compared to attractive angry and unattractive happy faces in both experiments. As we used different target faces in the two studies, we believe it to be unlikely that happiness was expressed more strongly in attractive faces and anger more strongly in unattractive face expressions. To us, it seemed more plausible that influences of attractiveness that were observed in the mood judgements in the MoC task also transfer to the emotional intensity ratings of the faces pictures. Future studies should aim to incorporate material in which attractive and unattractive faces express respective emotions to the same degree. One possibility would be to use computer-generated faces that are closely matched in terms of emotionality, gender and attractiveness. Another option would be to use machine learning approaches to select attractive and unattractive faces that show the same emotional intensity.

A second limitation is that we did not investigate participants’ own attractiveness, which may also play a role when judging the mood of attractive and unattractive crowds. Previous studies suggest that the degree to which individuals perceive another person’s attractiveness is strongly influenced by their own attractiveness (Sim, Saperia, Brown, & Berinieri, 2015). Therefore, future investigations could address participants’ attractiveness as an additional moderator. Especially individuals who perceive themselves as highly attractive might judge attractive crowds more favorably compared to unattractive crowds whereas this might not be the case for persons who rate themselves as being less attractive.

Lastly, it would be interesting to further investigate whether the strength of the effect of attractiveness on emotion perception is influenced by attractiveness-related stereotypes. It might be possible that the attractiveness effect is potentiated for participants who hold the attractiveness stereotype to a stronger degree (e.g. participants who evaluate attractive individuals more favorably compared to unattractive ones and associate attractiveness with more beneficial outcomes). Moreover, it would also be interesting to add an additional social cue to the current experimental design and systematically investigate the interplay between attractiveness and this social cue. For example, further varying—instead of experimentally controlling for— target gender in the experiment (by showing only female and only male crowds) would enable to test the interaction between target attractiveness and target gender on emotion perception in the mood-of-the-crowd paradigm. The attractiveness effect could be even stronger in female compared to male crowds as females are perceived more favorably compared to males (Eagly, Mladinic, & Otto, 1991a, b). However, this would require face databases with a larger number of male and female faces and with a greater variance of attractiveness to build the respective crowds.

## Conclusion

In two experiments, we demonstrated that attractiveness affects emotion perception in a visual search paradigm with multiple emotional targets, the MoC paradigm. Specifically, participants evaluated attractive crowds containing more happy expressions faster and more accurately, and the same was true for unattractive crowds dominated by angry expressions, which corroborates the evaluative congruence account. Moreover, attractive crowds were judged as being happy more often whereas unattractive crowds were perceived as being angry more frequently. Additionally, eye-tracking analyses revealed that there is also a small effect of attractiveness on gaze movements, though this was present only in experiment 1. Specifically, we observed higher fixation rates on happy compared to angry attractive targets, implying that attractiveness plays a role even in the early stages of perception. These results imply that face attractiveness should be carefully considered when selecting material for future studies that examine emotion perception, and stimulus attractiveness should be experimentally controlled or statistically adjusted for.

## Data Availability

Data from the experiments can be downloaded from https://heidata.uni-heidelberg.de/dataset.xhtml?persistentId=doi:10.11588/data/LYWYGN

## Footnotes

1 When conducting the power analysis for our studies we used the studies by Lindeberg et al. (2019) as a basis. They predominantly found medium effect sizes for the interaction effect between emotional expression and attractiveness regarding accuracy and categorization time across their four experiments. Although the task is not entirely comparable to the MoC task, we perceived this effect size to be the best anchor for our studies.

2 The norming data of the Chicago Face Database includes mean attractiveness ratings of 1087 participants (Ma et al., 2015) for each face picture. Because only the averaged ratings of each picture are available, the selection is based descriptively on these mean attractiveness ratings. Therefore, we report the mean and standard deviation averaged for the selected pictures of the respective types (female attractive, female unattractive, male attractive, male unattractive).

3 We chose the composition of the number of happy/angry faces based on the last experiment by Bucher and Voss (2019) which also included four trial types (7, 9, 11, 13 angry expressions in crowds of 20 faces). However, because we wanted to balance target gender in our crowds of faces, we needed to build crowds of even numbers of angry and happy expressions. Therefore, we changed the total number of pictures from 20 to 18 and changed the number of presented angry faces to 6, 8, 10 and 12.

4 To ease interpretation of the results, trial types containing more happy faces (6 and 8 angry out of 18 faces) and those who consisted of more angry faces (10 and 12 angry out of 18 faces) were combined. When analyzing the data using the original four categories of trial type, findings were largely identical.

5 When conducting the post-hoc analyses to shed light on this interaction between all factors, no meaningful interpretation of this effect could be revealed. Thus, this interaction effect does not contribute to the other findings from our analyses.

6 We re-ran all analyses and included the difference scores of the emotional intensity ratings for happy attractive and happy unattractive expressions (happy attractive minus happy unattractive) as well as for angry attractive and angry unattractive expressions (angry attractive minus angry unattractive) to account for this influence on our reported findings. However, including these difference scores did not alter the effects. There was still a significant effect of attractiveness on response tendency, *F*(1, 30) = 32.25, *p* < 0.001, *η*^2^_p_ = 0.52, 95% CI [0.29, 0.65], a significant interaction between attractiveness and dominant emotion on response time, *F*(1, 30) = 5.95, *p* = 0.021, *η*^2^_p_ = 17, 95% CI [0.01, 0.35], a significant interaction between attractiveness and dominant emotion on accuracy, *F*(1, 30) = 32.25, *p* < 0.001, *η*^2^_p_ = 0.52, 95% CI [0.29, 0.65], and a significant interaction between attractiveness and target emotion on number of fixations, *F*(1, 30) = 4.38, *p* = 0.045, *η*^2^_p_ = 0.13, 95% CI [0.00, 0.31].

7 Although the emotion ratings for the neutral expressions do not necessarily match the ratings of the actual emotions, it nevertheless provides a tendency that might influence the intensity of the emotional expressions.

8 We used the same power analysis criteria for the second experiment, to allow comparability between the two experiments.

9 There were several reasons why we decided to recruit a separate sample to rate the attractive and unattractive emotional face expressions. In the first experiment, ratings were collected after the MoC experiment, so it might be possible that the completion of the experimental task influenced the attractiveness and emotion ratings afterwards. Furthermore, when measuring the ratings prior to the experiment, it might happen that the ratings influence the completion of the experimental task afterwards which is also not ideal. Collecting the ratings in a “pretest” therefore seemed appropriate.

10 The findings with regard to participants’ gender need to be interpreted with caution as ratings from only 12 men were available.
